# Bioimprinted polymer platforms for cell culture using soft lithography

**DOI:** 10.1186/s12951-014-0060-6

**Published:** 2014-12-30

**Authors:** Lynn M Murray, Volker Nock, John J Evans, Maan M Alkaisi

**Affiliations:** The MacDiarmid Institute for Advanced Materials and Nanotechnology, Department of Electrical and Computer Engineering, University of Canterbury, Christchurch, 8140 New Zealand; The MacDiarmid Institute for Advanced Materials and Nanotechnology, and Centre for Neuroendocrinology, Department of Obstetrics and Gynaecology, University of Otago, Christchurch, 8011 New Zealand

**Keywords:** Bioimprint, Cell culture platform, Cancer cell, Soft lithography, Surface topography, Cell microenvironment

## Abstract

**Background:**

It is becoming recognised that traditional methods of culture *in vitro* on flat substrates do not replicate physiological conditions well, and a number of studies have indicated that the physical environment is crucial to the directed functioning of cells *in vivo*. In this paper we report the development of a platform with cell-like features that is suitable for *in vitro* investigation of cell activity. Biological cells were imprinted in hard methacrylate copolymer using soft lithography. The cell structures were replicated at high nanometre scale resolution, as confirmed by atomic force microscopy. Optimisation of the methacrylate-based co-polymer mixture for transparency and biocompatibility was performed, and cytotoxicity and chemical stability of the cured polymer in cell culture conditions were evaluated. Cells of an endometrial adenocarcinoma cell line (Ishikawa) were cultured on bioimprinted substrates.

**Results:**

The cells exhibited differential attachment on the bioimprint substrate surface compared to those on areas of flat surface and preferentially followed the pattern of the original cell footprint.

**Conclusions:**

The results revealed for the first time that the cancer cells distinguished between behavioural cues from surfaces that had features reminiscent of themselves and that of flat areas. Therefore the imprinted platform will lend itself to detailed studies of relevant physical substrate environments on cell behaviour. The material is not degraded and its permanency allows reuse of the same substrate in multiple experimental runs. It is simple and does not require expensive or specialised equipment. In this work cancer cells were studied, and the growth behaviour of the tumour-derived cells was modified by alterations of the cells’ physical environment. Implications are also clear for studies in other crucial areas of health, such as wound healing and artificial tissues.

## Background

Understanding the control of cell growth and proliferation are central to many health issues, including treatment of cancer [1], implantation of artificial tissues [2], and wound repair [3]. The role of the microenvironment is now well-recognised. In this regard a number of studies have investigated interaction of cells with substrates *in vitro*. Substrate modification has included the plating of small molecules or macromolecules, sometimes applied in patterns. For example molecularly imprinted polymer studies have been undertaken with proteins [4] and an independent role for topography has been suggested [5]. Advances in nanotechnology, such as nanoimprint lithography [6,7], produce topographical surface features down to the nanometre scale and allow for investigation of biomaterial interfaces without chemical variation. Topographically-modified substrates, with wide ranging pattern magnitudes and geometries, have been shown to affect the growth characteristics of cultured cells [8,9]. This hypothesis has led to a number of investigations involving manufacturing physical patterns on substrates in the form of pits, pillars or gratings. These structures are often of smaller dimensions than those of the cells that would constitute a physiological neighbourhood and the relevance of these structures to *in vivo* conditions is uncertain. While these geometric patterns have provided substantial pointers to the importance of the physical environment, they do not contain features that would be recognised by a cell *in vivo*. In this study we report development of a method that replicates cell shapes in a polymer and thus contains features of similar size and shape to that of a cell’s microenvironment.

We employed Bioimprint methodology [10] in this study. This technique is inspired by nanoimprint lithography and was initially developed in our studies to circumvent deficiencies in high-resolution live cell imaging. Atomic force microscopy (AFM) imaging of live cell cultures was difficult due to the elasticity of the cell membrane and electron microscopy techniques require sacrificial cell samples. A replication protocol was developed to mould the cell surface features into a more rigid and tear-resistant material. The resulting methacrylate co-polymer imprint contained high resolution cell-like features, accurate to 5–20 nm [11-14]. Although other groups have investigated the use of polymeric imprints of cells to obtain information on cell morphology [15,16] this study extends the methodology to enable investigation of cell function.

In this study the biocompatibility of the polymer is confirmed, and we have adapted the imprinted polymer for use as a cell culture platform. We demonstrated a preferential adherence of the cells for the imprinted regions compared to flat areas. These biocompatible bioimprinted templates will provide a platform with potential for investigating localised variation and specific cell adhesion.

## Results and discussion

### Bioimprint substrates

Bioimprint is a technology we developed for replicating biological cells at high resolution in hard polymer for the purpose of imaging or formation of cell culture platforms. To produce an imprinted substrate it was necessary that the substrate for this initial culture could be separated from the cured polymethacrylate in which the initial culture was moulded. Hence glass was chosen for the initial substrate. Glass provided good cell adhesion and growth environment with minimal adhesive interaction to cured methacrylate co-polymer. Polystyrene, a common surface for cell culture, was not suitable substrate for the initial culture because it formed an inseparable adhesive bond with the cured polymethacrylate.

**Figure 1:**
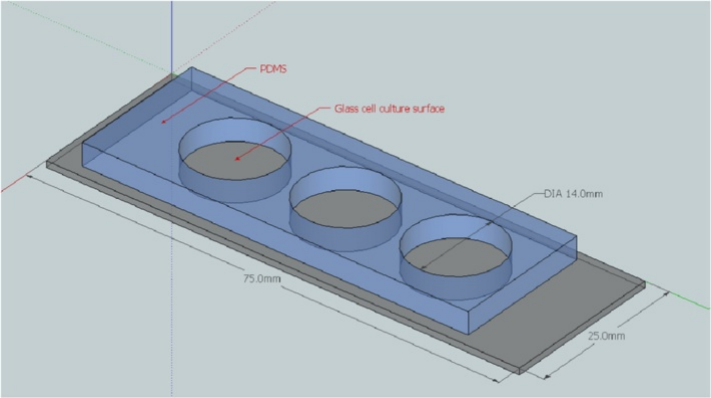
**Polydimethylsiloxane [PDMS] with 14 mm circular cut-outs conformally sealed to a glass microscope slide for use as a cell culture substrate for the bioimprinting protocol.**

PDMS-defined borders on culture wells were found to be ideal for confining cultures because of their inexpensive and fast fabrication, adaptability to different size requirements, the reversible but stable conformal seal of PDMS to glass, and fabricated assemblies could be autoclaved to maintain sterile conditions in culture. Circular chamber structures (Figure 1), as opposed to rectangular chambers, were found to minimise the stress induced on the polymethacrylate during UV curing. Due to the short, high intensity UV exposure, chamber designs containing corner regions showed increased mechanical stress in those regions and induced a concavity across the substrate.

The optimal ratio for the liquid methacrylate co-polymer mixture was determined to be 600 μL EGDMA: 300 μL MAA: 100 μL IRGAcure 2022 because of the balance required between the optical and stress properties. More equal ratios of the monomer groups produced a cloudy to opaque white polymer depending on the monomer concentration. Larger ratios (as similar as 600:200:100) caused fatal cracking during the curing phase due to the increased relative quantity of EGDMA cross-linker.

Using the optimised ratio, bioimprint substrates consistently cured into rigid, transparent substrates which were easily separated from the underlying glass microscope slide used for initial cell culture. For assurance of complete curing, chambers containing liquid pre-polymer were exposed to UV for 240 seconds but most bulk curing was complete after as little as 30 seconds.

**Figure 2:**
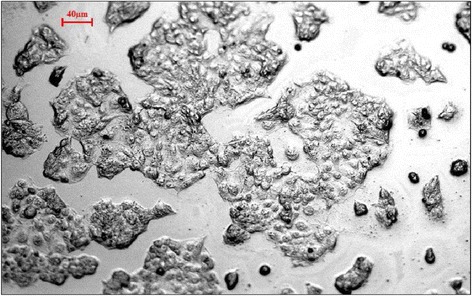
**Differential interference micrograph showing a bioimprinted substrate surface containing replica features of Ishikawa cells in polymethacrylate.**

**Figure 3:**
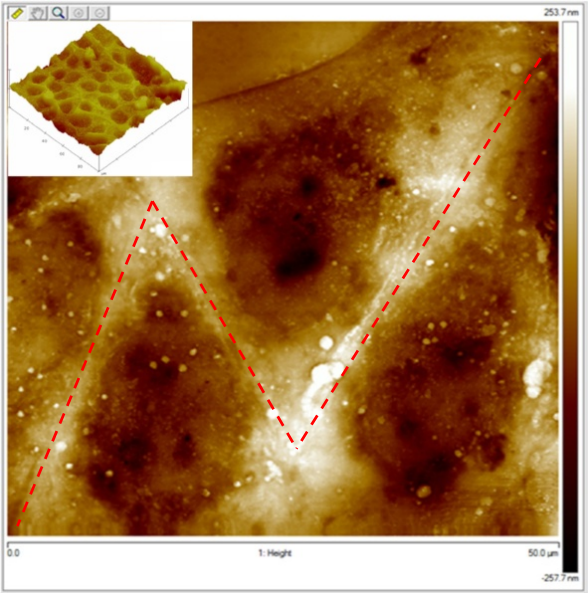
**Atomic force microscopy image of multiple cells showing the replication fidelity of bioimprinted Ishikawa cell features in polymethacrylate.** Because it is a negative mould indentations and pores on the cell surface appear as protrusions on the AFM. Similarly, the nuclear envelope appears as an impression into the polymer surface. Insert: Low magnification AFM image of Bioimprint in polymethacrylate of a culture of Ishikawa cells. Red dotted lines identify cell borders, dark areas are the replicated nuclei.

The bioimprinting protocol successfully produced high resolution replicas of Ishikawa cell features into permanent polymer substrates. Using a pipetting application method instead of spin-coating, which consequently allowed for the removal of triglyme as a thickening agent, did not appear to affect the replication resolution of the methacrylate. Differential interference contrast (DIC) (Figure 2) and AFM (Figure 3) showed there was high fidelity feature replication where micron and nanometre scale details are evident.

### Biocompatibility

**Figure 4:**
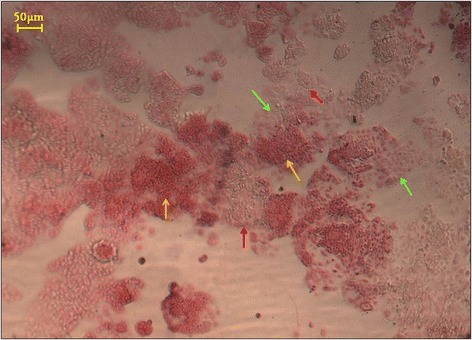
**Cell cultured on bioimprinted platform with cell like features.** Eosin-stained Ishikawa cells grown for 24 hrs on bioimprinted substrates taken at 24 hrs of initial culture (shown at 50x magnification). Arrows indicate areas of high density cell growth (yellow), cell growth away from bioimprinted regions on flat areas of substrate (green), and identifiable bioimprint regions which are not coverd by cells of the secondry culture (red).

Bioimprint substrates, with and without triglyme, diffused acidic solutes beyond the limiting capacity of the sodium bicarbonate buffer of the cell culture medium. The substrates were therefore subjected to different washing treatments prior to immersion in α-MEM. The phenol red pH indicator included in the medium revealed residual acid in wells in which the substrate had been washed only once with α-MEM prior to immersion. Medium of bioimprint substrates that had been washed with both deionised water and α-MEM leached less acid. Substrates washed with 0.1 M NaOH in addition to deionised water and media washes had no effect on the phenol red pH indicators and thus were satisfactory as culture substrates. Further, methacrylate substrates including triglyme affected pH only slightly less than substrates cured from mixtures not containing triglyme. An extended water wash (>24 hrs) was added to the protocol to provide a tolerance step. This was followed by an additional wash in fresh, sterile medium prior to the application of cells in medium to the substrate. Excluding triglyme from the polymerisation mixture improved optical translucency and the elasticity of the cured polymer substrate. Cells were successfully grown on the prepared platforms as shown in Figure 4.

### Secondary cell culture

When cells were incubated on imprinted surfaces the cultured cells exhibited different attachment and growth on the Bioimprint patterned substrate surface compared to those cells on areas of flat surface as shown in Figure 4. Thereby it was revealed that cells distinguish surfaces that had features reminiscent of themselves. The observation therefore indicated that the physical nature of the substrate influenced the cells’ behaviour.

**Figure 5:**
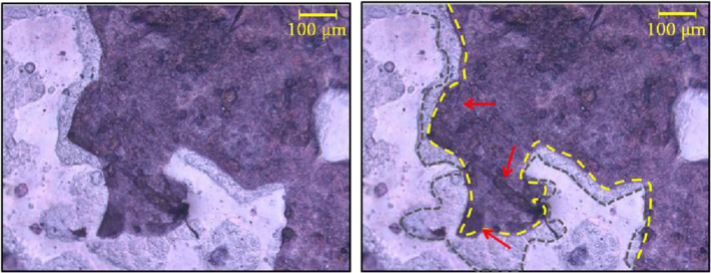
**Ishikawa cells grown on bioimprinted substrates for 48 hours, cells are stained with Commassie blue.** Left image, unannotated. Right image, the bioimprint features are outlined in grey, the secondary cell culture (dark blue) is outlined in yellow. Arrows (red) note regions of the cell monolayer that peeled off and folded back. This figure illustrates that the growth of the secondary cell culture has been guided by the footprint of the replicated cell patterns.

It was observed that in cultures that had longer period of proliferation (48 hrs) some of the periphery of the cell culture had lifted during staining. We were able, in these cases, to note that growth of the cells followed the pattern of the imprinted areas (Figure 5). Coomassie Brilliant Blue staining clearly shows cells adhering and spreading across the bioimprinted surface and adhering preferentially to the imprinted surface. Therefore the polymethacrylate substrate will lend itself to detailed studies of behavioural cues generated by relevant physical environments. It is remarkable to observe for the first time how cells reacted to patterns that resemble themselves by following the footprint of the bioimprinted features.

**Figure 6:**
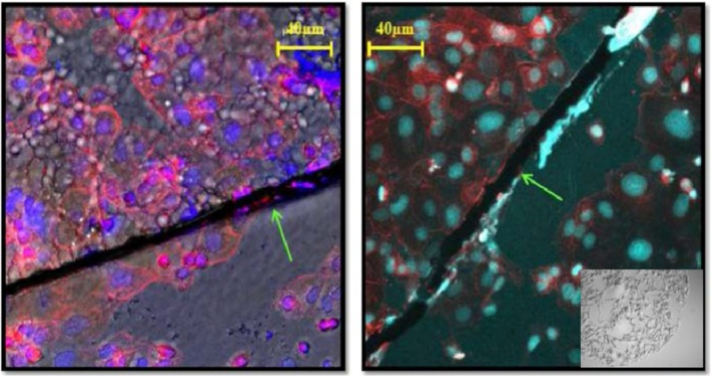
**Stencilled Ishikawa Bioimprints showing designated regions of bioimprint features.** Left: Confocal fluorescence image of counterstained Ishikawa cells growing on stencilled substrates. Blue, nuclei; Red, cytoplasm. Right Confocal imaging using bright field background channel (which allowed for a single focal plane). Green arrows indicate the border between the flat and bioimprinted (upper) areas and flat (lower) areas. Insert showing a wide view of the border between the flat and imprinted areas of a stencil.

The differential attachment was confirmed by manufacturing imprinted patterns in defined areas using stencils. By this means whether the cells were on flat or imprinted areas could be readily determined by their localisation within the chamber. The results (Figure 6) indicated that cells preferentially adhered and grew on imprinted areas and grew closer to each other.

Lithographically defined micro patterns of pillars and holes were prepared on platforms and cells were cultured on these substrates for comparison with the bioimprint. The dimensions of the lithographically defined patterns were chosen relative to typical Ishikawa cell size which is between 10–50 microns.

**Figure 7:**
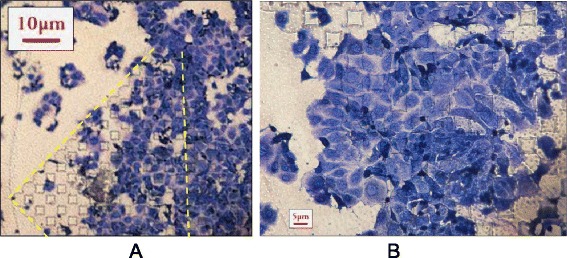
**Ishikawa cells grown on substrates with lithographically-manufactured patterns of similar overall size, cells are stained with Commassie blue.** **(A)** - illustrates an array of 5 µm diamond shaped pillars prepared on polymethacrylate substrate, Ishikawa cell were cultured and stained with Coomassie blue. The cells show no evidence of preferential spread between patterned and flat regions; **(B)** is a high magnification image.

Low magnification bright field imaging showed groups of cells clustered across the lithography manufactured substrate, but there was no evidence for differential preference between patterned and unpatterned surfaces (Figure 7). This is in contrast to the observation using Bioimprints. Figure 7 illustrates an array of 5 μm diamond shaped pillars prepared on polymethacrylate substrate; Ishikawa cells were cultured and stained with Coomassie blue. The lithographic patterns without cell-like features had no effect on cell spread between patterned and flat regions.

Interest in topography as an influence on cell behaviour and thence potentially on a range of aspects of health and treatments, independent of biochemical factors, has recently increased. Thus it is important to obtain information on the contribution of cell environment to pathologies such as cancer, as studied in this project, and vascular disease and to interventions that include implants and wound repair.

To improve the effectiveness of inserted medical devices which is expected to be used for monitoring, detections and diagnostics of our health status the interface between the body and foreign materials must be examined and characterised. However we noted the absence of models for *in vitro* investigations that incorporated physical environments similar to those experienced *in vivo*. Here we have, for the first time, developed solid, robust substrates that have features similar to the cells being studied.

Mixed methacrylate co-polymer was able to reliably replicate high resolution Ishikawa cell surface features to less than 50 nm through fast, high intensity UV exposure methodology [17]. We have used Bioimprints directly for cell imaging studies previously [10,18]. In this study, modifications to the Bioimprint pre-polymer composition and protocol (i.e. removal of triglyme and improving its biocompatability) allow bioimprinted samples to be used as cell culture platforms.

The high light intensity required for the fast curing produced two notable effects on the curing polymer: heat generation and induced stress. Heat generation did not alter the bioimprint quality unless the refrigerated templates of fixed cells were not allowed to acclimate to room temperature prior to exposure and thence introduce defects, possibly resulting from bubbles at the bioimprint interface. The problem of induced crosslinking stress was minimised by using circular sample geometry instead of rectangular geometry. In the chosen geometry the radial distribution of stress induced by high speed (30 seconds), high intensity (100w) UV source curing minimised the curving of the polymer and improved planarization of the bioimprinted surfaces.

The Bioimprint provides a simple and readily-adaptable platform to investigate cell behaviour by methods commonly used for traditional *in vitro* cell culture. The method produces a substrate with nanometre resolution of cell surface features that has attributes that are not provided by the soft surfaces of other cell imprinted templates [19,20]. Importantly the topography-related structures, obtained by the overlay imprinting of the method described here, are more comprehensive than, for example, those obtained from tissue sections [21]. We observed adherence and growth patterns of the cancer cells on imprinted areas that were distinct from behaviour on flat polymethacrylate surface. Growth is recognised as occurring in areas on the culture platform where cell viability and attachment is high. These results indicate that the cells identified differences in physical topography (flat compared to imprinted) since the substrates, being on the same culture chamber, had been treated identically. We suggest that investigations of guiding cell growth in areas that are currently receiving extensive attention, such as stem cells development and tissue engineering, will also benefit from the method. Other advantages are the ability to store cell details in a hard polymer and prepare cell culture platforms for controlled cell behaviour.

The Bioimprint methodology provides a means of studying cell behaviour in a physical environment that has features of the order of those found *in vivo* and provides a three-dimensional component to the cells’ environment. This development is a step increase in biomimicry over that provided by geometrically manufactured substrates. It will be possible, when technical issues are optimised, to extend the concept to imprints in other formats such as cells replicated with structures convex to the base, flexible substrates and in a variety of polymers. It is likely to become possible to manufacture a series of identical substrates from a master mould so that pharmacological treatments of cells on the same imprinted structures can be undertaken. Additionally bioimprinted surfaces may be modified using techniques already developed such as with protein [4] or DNA [22], or adapted to be employed with particulate entities such as viruses [23] to further increase their functionality.

The method produces relevant topography in relation to a cell’s micro and nanoenvironment *in vivo*. The resolution of the chosen polymethacrylate polymer is very high (nanometre) and the role of these features that are replicated at this level remain to be defined. The polymer with imprint is permanent and so can be potentially reused within an experiment, incorporated into a later study, or shared with other research laboratories. The process is easy, requires only simple equipment, is inexpensive and the substrate does not require molecular modification. Thus the method provides unique platforms on which the effects of the physical shapes and topography can be investigated. The role of mechanotransduction, the effects on cell behaviour of altered morphology, the cues by which the physical environment either induces tumorigenesis or maintains homeostasis in cells, can all be subjects of study using this method. Importantly this study reported observations on cancer cells of morphological alteration and differential adherence characteristics induced by cues provided by culturing cells on flat and on bioimprinted cell-like patterned platforms.

Determining the effects of the micro-scale patterns allowed us to separate observations of cell growth on flat, micro-patterned and bioimprinted substrates including the nano-scale topographical features. When cells were cultured on the lithographically defined substrate, the pattern showed no effect on the overall culture organization and growth. Cell clusters were visible across the diameter of the substrate irrespective of whether there are patterns or not.

## Conclusions

We report development of a unique technique for printing a biological cell in hard polymer that provides high resolution replication and offers a cell culture environment with cell-like features. This enabled us to observe for the first time how cells develop growth characteristics in response to an environment patterned with features that resemble themselves. This methodology has high potential for applications in tissue engineering, medical implants and in studying the influence of physical environment on cell behaviour.

## Methods

### Cell culture protocol

Ishikawa endometrial cancer cells were cultured in circular chambers of polydimethylsiloxane (PDMS) on glass substrates. To fabricate the PDMS wells, liquid PDMS (Dow Corning, Midland, MI) was mixed at 10:1 elastomer to cross linker ratio, stirred thoroughly, and deaerated before curing. PDMS was poured into polystyrene dishes, which were levelled on a hot plate for curing at 80°C for 2 hrs. Circular chambers were punched into cured PDMS sheets using a 14 mm cork borer. PDMS sheets were then cut to fit a microscope slide and conformally sealed to the slide. PDMS/glass slide constructs were sterilised before use as cell culture substrates.

Ishikawa cells were seeded into the PDMS-bordered wells at 5.0 × 10^4^ cells/cm^2^ in α-minimum essential medium (α-MEM) supplemented with 2.2 g/L sodium bicarbonate, 10% fetal bovine serum, 1% GlutaMAX, and 1% penicillin/streptomycin. Ishikawa cells were incubated at 37°C and 5% CO_2_ for 24 hrs before medium was aspirated and replaced with 4% paraformaldehyde in PBS for at least 30 minutes for cell fixation prior to bioimprinting (all purchased from Life Technologies Co., Carlsbad, CA). Fixative was removed and cultures were rinsed thoroughly in separate PBS and water washes before being placed in 4°C storage for at least 2 hrs to encourage drying of excess water before bioimprinting. Fixed Ishikawa cell cultures were removed from refrigerated storage prior to polymer mixing to bring the samples to room temperature before UV exposure to minimise condensation and bubble artefacts at the cured bioimprint-cell interface.

### Bioimprint substrates

The liquid methacrylate co-polymer used for bioimprint substrate fabrication was adapted from previous work [18]. Ethylene glycol dimethacrylate (EGDMA) and methacrylic acid (MAA) (both purchased from Sigma Aldrich, St. Louis, MO) were mixed at the optimised ratio of 600 μL to 300 μL with ~100 μL IRGAcure 2022 (CIBA Specialty Chemicals Basel, Switzerland) added as a photoinitiator. Triglyme was added to the mixture as a thickening agent. The liquid methacrylate solution was mixed for at least 30 seconds with a vortex mixer before being pipetted into the PDMS-defined cell culture wells. The liquid polymer solution was allowed to settle for 10–20 seconds before UV exposure to ensure maximum resolution of small-scale cell features. Slides were placed 15 cm directly beneath a UV light (Omni Cure series 1000 UV, 100w Hg arc lamp, 250-450 nm filter, EXFO Photonic Solutions Inc. Singapore) guide and exposed to UV at 40% aperture opening for 240 seconds. Cured imprints were removed from the PDMS/glass assembly to a water bath and manually agitated to remove larger cell debris. The cured bioimprint was then transferred to an ultrasonic sodium dodecyl sulphate bath (10% w/v in .01 M hydrochloric acid solution) and a 30 minute trypsin soak (0.05% trypsin in PBS) in order to minimise cell material remaining on the bioimprinted polymer surface.

### Patterned substrate fabrication

To directly compare the effects of geometrically patterned lithography with those of the bioimprint, different patterned substrates were fabricated. Patterned substrates consisted of regular geometric arrays of pillar or hole patterns of 5–15 μm comparable to the size of the cells under study. The patterns were initially fabricated in SU-8 photoresist (MicroChem SU-8 2100) on silicon wafers using photolithography processes and inverse PDMS moulds were made using soft lithography. The PDMS patterned platforms were replicated in polymethacrylate substrates for cell culture experiments. Ishikawa endometrial cancer cells were cultured in the same conditions as the bioimptinted platforms.

### Biocompatibility

To neutralise leaching of methacrylic acid the quenching effect of different washing techniques on the polymethacrylate (EGDMA) substrates prior to use in cell culture was investigated. Bioimprint samples were placed in 12 wells of a 24-well polystyrene tissue culture plate. Bioimprints were washed with (i) deionised water followed by α-MEM medium, (ii) only α-MEM medium, (iii) 0.1 M NaOH followed by deionised water and α-MEM medium, or (iv) left untreated. Washes were pipetted into each well, agitated for approximately 30 seconds, and aspirated. After removal of wash conditions, each well was filled with fresh α-MEM (without cells present) containing phenol red pH indicator.

Cytotoxicity of bioimprinted polymethacrylate samples was investigated by placing a cured bioimprint substrate at the bottom of 3 wells on a polystyrene 6-well plate; the 3 wells without bioimprint samples were maintained as control cultures. Ishikawa cells were seeded at 5.0 × 10^4^ cells/well in all 6 wells and incubated in accordance with the previously outlined protocol for 24 hours, at which point the substrates were removed for imaging.

### Secondary cell culture

Ishikawa cells were grown on bioimprinted substrates to verify the biocompatibility of the substrate and determine the topographical influence of bioimprinted features on cell attachment and growth. Ishikawa cells were seeded and cultured on bioimprinted polymethacrylate substrates placed on the bottom of 24-well polystyrene plates. These cells were referred to as secondary cell cultures in order to distinguish them from the initial cell cultures required for bioimprint substrate fabrication. Bioimprints were placed template-side-up and Ishikawa cells were seeded at 5.0 × 10^4^ cells/well and maintained in supplemented α-MEM at 37°C and 5% CO_2_ for 24 hrs. At 24 hrs medium was aspirated and cells were fixed with 4% paraformaldehyde in PBS for at least 30 minutes and then washed with PBS several times to remove trace fixative and salts. Cells were stained with Coomassie brilliant blue (Life Technologies Co., Carlsbad, CA) for 5 minutes and washed at least twice with PBS until wash solutions did not contain leached stain.
